# How Early is Infants' Attention to Objects and Actions Shaped by Culture? New Evidence from 24-Month-Olds Raised in the US and China

**DOI:** 10.3389/fpsyg.2016.00097

**Published:** 2016-02-05

**Authors:** Sandra R. Waxman, Xiaolan Fu, Brock Ferguson, Kathleen Geraghty, Erin Leddon, Jing Liang, Min-Fang Zhao

**Affiliations:** ^1^Department of Psychology and Institute for Policy Research, Northwestern UniversityEvanston, IL, USA; ^2^Institute of Psychology, Chinese Academy of SciencesBeijing, China; ^3^Department of Psychology, Northwestern UniversityEvanston, IL, USA; ^4^Department of Linguistics, Northwestern UniversityEvanston, IL, USA

**Keywords:** infants, attention, culture, dynamic events, actions, objects, china, united states

## Abstract

Researchers have proposed that the culture in which we are raised shapes the way that we attend to the objects and events that surround us. What remains unclear, however, is how early any such culturally-inflected differences emerge in development. Here, we address this issue directly, asking how 24-month-old infants from the US and China deploy their attention to objects and actions in dynamic scenes. By analyzing infants' eye movements while they observed dynamic scenes, the current experiment revealed striking convergences, overall, in infants' patterns of visual attention in the two communities, but also pinpointed a brief period during which their attention reliably diverged. This divergence, though modest, suggested that infants from the US devoted relatively more attention to the objects and those from China devoted relatively more attention to the actions in which they were engaged. This provides the earliest evidence for strong overlap in infants' attention to objects and events in dynamic scenes, but also raises the possibility that by 24 months, infants' attention may also be shaped subtly by the culturally-inflected attentional proclivities characteristic of adults in their cultural communities.

## Introduction

Do the cultures in which we live shape the way that we view the objects and events in the world that surrounds us? This question, which has captivated curiosities for centuries, engages fundamental questions about which human capacities (if any) are universal and how they are shaped by experience. Within psychology, linguistics and anthropology, researchers have tackled this question by focusing on capacities as diverse as color perception, moral reasoning and decision-making (see Haidt, 2001; Özgen, 2004; Atran et al., 2005, for reviews of these capacities, respectively). Within the developmental sciences, researchers have advanced these lines of inquiry by identifying how early in life our most basic perceptual and cognitive systems begin to show the effect of the cultures in which we are raised (Bornstein, 2010).

Consider, as a case-in-point, the evidence for cultural differences in the ways we direct our attention to the objects and events that surround us. Decades of research suggest that when observing scenes, adults from the US focus predominantly on objects, while those from China and Japan direct more of their focus to the contexts and events in which those objects are engaged (c.f., Nisbett et al., 2001; Chua et al., 2005). For example, when adults from the US and Japan were asked to describe a dynamic scene from an aquarium, those from the U.S. primarily described a large fish located at the center of the scene, yet those from Japan described the same fish in the context of the actions in which it was engaged (e.g., swimming) and its relation to other entities in the scene (e.g., the plants and smaller fish around which it was swimming) (Masuda and Nisbett, 2001). More recent evidence suggests that such differences likely arise from cultural differences in basic attentional patterns. Chua et al. (2005) measured the eye movements of American (US) and Chinese adults while they viewed photographs with a focal object on a complex background. Americans not only fixated more on focal objects than did the Chinese, but also tended to look at the focal object more quickly. In contrast, the Chinese devoted more attention to the background than did the Americans. Differences like these have been documented in a variety of tasks that tap into adults' perceptual, social, and reasoning capacities (Ji et al., 2000; Masuda and Nisbett, 2001, 2006; Nisbett et al., 2001; Kitayama et al., 2003; Nisbett and Masuda, 2003; Chua et al., 2005; Nisbett and Miyamoto, 2005; Richland et al., 2007; Masuda et al., 2008; see Imai and Masuda, 2013, for a broader review). Moreover, these culturally-guided differences have been documented in children as young as 3 or 4 years of age in capacities as diverse as attention, cognition and language (Imai et al., 2005, 2008, 2010; Saalbach and Imai, 2007; Lockhart et al., 2008; Duffy et al., 2009; Hanania and Smith, 2010; Richland et al., 2010; Göksun et al., 2011; Kuwabara et al., 2011; Kuwabara and Smith, 2012; Moriguchi et al., 2012; Imada et al., 2013).

This raises a new developmental challenge: If we are to discover when culture-specific patterns of attention begin to shape our outlook on the world, we must set our sights earlier to infants in the first years of life. There is already reason to suspect that infants' attention to objects and events in dynamic scenes might already be influenced by culture-specific patterns of attention. We know, for example, that infants attend carefully to the actions of their parents and other important others, and that by 7 months, they follow their tutors' eye-gazes and pointing (Senju and Csibra, 2008). Moreover, certain patterns of attention are woven inextricably in our interactions with infants: Parents from the US tend to engage their infants in games and social routines that emphasize objects over actions and relations, while parents from China and Japan tend to engage their infants in activities that emphasize actions over the objects themselves (Fernald and Morikawa, 1993; Tardif et al., 2008a). What remains unanswered is whether infants pick up on the distinct attentional patterns like these. Is their allocation of attention to objects and events in dynamic scenes shaped by the attentional proclivities of the adults in their respective cultural communities? Addressing this question requires comparing the attentional strategies of infants in the US and China.

With this goal in mind, we built a design based upon three well-established cornerstones. First, in the first year of life, even before infants begin to speak, they are able to form mental representations of *both* objects and events (Gordon, 2003; Baillargeon, 2004). Second, when infants observe dynamic scenes, they notice both the objects in these scenes and the actions in which they were engaged. For example, in one experiment, 20-month-old infants viewed videotaped dynamic scenes (e.g., a novel cartoon-like creature jumping back and forth over a fence); next they viewed this now-familiar scene, pitted against a new scene in which *either* the object changed (e.g., a new creature jumping over a fence) *or* the action changed (e.g., the same creature racing across a platform). Infants from three different cultural and linguistic communities—Japan, France and the US—detected changes of both kinds, as evidenced by their reliable preferences for the new (changed) scene. This suggests that they had the representational flexibility to attend to either the objects or the actions in these novel, dynamic scenes (Katerelos et al., 2011).

Third, by 24 months, infants take advantage of this representational flexibility when learning words: they tend to map novel nouns to objects and object categories, and novel verbs to actions and event categories (Fisher, 1996; Waxman et al., 2009; Maguire et al., 2010; Chan et al., 2011; Leddon et al., 2011; Oshima-Takane et al., 2011; Arunachalam et al., 2013; Chen and Waxman, 2013). For example, one series of experiments examined how monolingual 24-month-olds from either the U.S., Korea or China viewed a series of dynamic scenes (e.g., a girl petting a toy dog) while listening to sentences containing a novel word (Leddon et al., 2011; Arunachalam et al., 2013). For some infants the novel word was presented as a noun (e.g., “The girl is petting the *blick*”); for others, it was presented as a verb (e.g., “The girl is *blicking* the dog”). At test, infants viewed two new scenes simultaneously. One involved a change in the object but maintained the same action (e.g., the girl petting a pillow), the other involved a change in the action but maintained the same participant object (e.g., the girl kissing the dog). With the two test scenes in full view, infants were asked to choose between them by pointing. Infants in the Noun condition heard, e.g., “Where is the *blick*”? Those in the Verb condition heard, e.g., “Where is she *blicking* something?” At issue was whether infants' choice of test scenes was influenced by the kind of word they were learning. The answer was a resounding “yes.” Infants from all three countries revealed the same pattern: Infants who had been introduced to novel nouns pointed toward the scenes that maintained the same object (e.g., dog), despite the fact that it was now engaged in a different action (e.g., the girl *kissing* the dog). In contrast, infants who had been introduced to novel verbs pointed to the scene that maintained the same action (e.g., petting), even though it now involved a different object (e.g., the girl petting a *pillow*).

Still, even with these three cornerstones firmly in place, a key question remains unresolved: How do infants deploy their attention when they are simply observing dynamic scenes as they unfold? This is a serious gap because if we are to identify whether, and under what circumstances, novel words direct infants' attention toward either objects or events, we must first understand how infants, from across the world's cultures, deploy their attention in a baseline task, one that does not also involve word learning. When infants are simply observing dynamic scenes, does their attention bear the imprint of their respectively “object-oriented” or “action-oriented” cultural communities?^1^ Answering this question requires that we move beyond infants' pointing as a dependent measure, employing instead state-of-the-art eye-tracking paradigms to trace how infants direct their attention in real time as the dynamic scenes unfold.

Our goal in the current experiment was to fill this gap. We recruited 24-month-old infants raised in either China (Beijing) or the US (Chicago) to assess how they deploy their visual attention to objects and events in dynamic scenes. We adapted a robust experimental paradigm, originally designed to identify infants' expectations for extending newly-learned nouns and verbs. We know that in this task, 24-month-old infants extend novel nouns to scenes that include the same object (e.g., dog), even if it is now engaged in a different action (e.g., the girl *kissing* the dog), and that they extend novel verbs to scenes that include the same action (e.g., petting), even if it now involved a different object (e.g., the girl petting a *pillow*) (Waxman et al., 2009; Arunachalam and Waxman, 2011; Leddon et al., 2011). Here, we step back, modifying the design in two key ways. First, we ask how infants from China and the US deploy their visual attention when they simply observe these dynamic scenes as they unfold, but are not simultaneously engaged in the task of word learning. Second, we use infants' visual attention to the scenes (eye-tracking), rather than pointing, as a dependent measure. We focused on 24-month-olds, an age at which infants in both the US and China are just beginning to produce verbs. This permitted us to assess whether infants from the US and China—who are in the midst of learning words for objects and actions—share the same attentional strategies or whether their attention might be shaped already by the culturally-inflected proclivities characteristic of adults in their respective communities.

## Methods

### Participants

Forty monolingual 24-month-old infants participated. The US infants (*N* = 20; 10 males; *M*_age_ = 23.86 months, range 23.03–25.33) were recruited from Evanston, IL and its surrounding communities. The Chinese infants (*N* = 20; 16 males; *M*_age_ = 23.89, range: 23.05–25.87) were recruited from Beijing, China. In the US, four additional infants were excluded for fussiness (*N* = 3) or experimenter error (*N* = 1). In China, six additional infants were excluded for fussiness. Infants from both countries were from primarily college-educated families in the middle- and upper-middle-class.

This study was carried out in accordance with the recommendations of the Northwestern University IRB and the Chinese Academy of Sciences IRB, with written informed consent from a parent of each infant. All parents gave written informed consent in accordance with the Declaration of Helsinki.

Parents also completed a standardized checklist of the words in their infants' productive vocabularies. Parents in the US completed the MacArthur Short Form Vocabulary Checklist (MCDI Level II Form A (Fenson et al., 2000); those in China completed the Chinese (Mandarin) Short Form Communicative Development Inventory (Putonghua) (PCDI) (Tardif et al., 2008b). Infants' vocabulary size in both the US (*M* = 62) and China (*M* = 78) were well within the standardized norms established for their respective countries. This provides assurances that our participants' language development was broadly representative of other infants in their respective communities.

### Materials

Please see Table 1 for the design protocol and a representative set of materials.

**Table 1:** **One representative trial in English (US) and Mandarin (China)**.

	**Dialogue**	**Familiarization Dynamic scenes, presented one at a time**						**Test Dynamic scenes, presented simultaneously**	
		**Exemplar Trials**				**Contrast Trials**		**New Object Familiar Action**	**New Action Familiar Object**
**Video Stream** → **Audio Stream 1** ↓	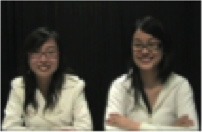	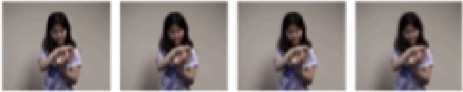				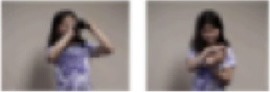		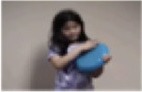	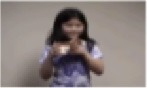
English (US infants)	(Dialogue in English)	*Wow! Look what's happening here*	*Look at this*	*Do you see that?*	*Hey! Look there*	*Uh oh! Look at that!*	*Yay! Look at this!*	Baseline: *Now look, they're different* Response: *Did you see it? Did you see it?*	
Mandarin (Chinese infants)	(Dialogue in Mandarin)	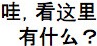	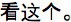	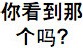	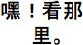	Uh-oh! 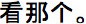	Yay! 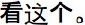	Baseline: 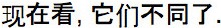 Response: 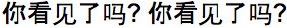	

*Each trial included a Dialogue Phase, Familiarization Phase, and Test Phase (including a Baseline and Response period)*.

#### Visual stimuli

Videos were digitized recordings of live actors, edited to create the sequences of scenes shown in Table 1. In the Dialogue scenes, two actors were seated next to each other, engaged in a dialogue. In the Familiarization and Test scenes, human actors performed continuous actions on inanimate objects. The actors, actions and object in these latter scenes were taken from Waxman et al. (2009). Infants in the US and China viewed the very same visual scenes; the accompanying linguistic information (e.g., “Look at this”) was presented in their native language (either English or Mandarin). All visual materials were presented on a Tobii T60XL eye-tracker monitor. Table 2 provides a complete list of the visual scenes we presented to infants.

**Table 2:** **Complete set of dynamic scenes presented on each trial**.

**Familiarization Scene**	**Test Scenes**	
	**New Object-Familiar Action**	**Familiar Action-New Object**
Girl1 petting (toy) dog	Girl1 petting frisbee	Girl1 kissing dog
Boy1 waving balloon	Boy1 waving rake	Boy1 tapping balloon
Girl2 twirling umbrella	Girl2 twirling pillow	Girl2 twisting umbrella
Boy2 pushing chair	Boy2 pushing box	Boy2 lifting chair
Girl3 washing cup	Girl3 washing plate	Girl3 drinking from cup
Boy3 pulling bunny	Boy3 pulling drum	Boy3 tossing bunny

#### Auditory stimuli

All auditory stimuli were recorded in a sound-attenuated booth by female native speakers of either American English or Mandarin who adopted an infant-directed speech register. The English stimuli were identical to those used in the No Word condition of Arunachalam and Waxman (2011) and Waxman et al. (2009). The Mandarin stimuli were translated from English to Mandarin by a native Mandarin speaker, bilingual in English, and then recorded. Our goal in the translation was to balance fidelity to the English stimuli with naturalness in Mandarin infant-directed speech. Recordings in each language were then edited to match one another in timing, duration, mean amplitude, fundamental frequency and pitch peaks. These auditory stimuli were then synchronized with the visual stimuli (Table 1) and presented in stereo from the Tobii T60XL speakers hidden below the monitor.

#### Apparatus and procedure

Infants and their caregivers were welcomed into a laboratory playroom; while the infants played freely with toys, caregivers completed the vocabulary inventory. Next, the experimenter escorted the infant and caregiver into an adjoining test room where the infant was seated with eyes positioned 60–70 cm directly in front of a 52 × 32 cm monitor screen. Infants sat either in an infant-seat or on the caregiver's lap. Caregivers wore opaque glasses^2^ (or closed their eyes) and were instructed not to talk during the experiment or to influence their infant's attention in any way. The experimenter then moved behind the monitor to control the experimental procedure (described below). Before beginning the experiment itself, we conducted a standard 5-point calibration procedure with the eye-tracker. Thereafter, and throughout the experiment, infants' eye gaze direction was sampled at a rate of 60 hz by the eye-tracker. Sessions lasted approximately 10 min.

#### Experimental task

The procedure itself included three distinct phases: dialogue, familiarization and test (Table 1). Each infant completed this three-phase procedure six different times (trials). Each trial involved a different dialogue followed by a different sequence of dynamic scenes (e.g., a girl petting a dog; a man waving a balloon). Table 2 provides a complete description of the scenes depicted in each trial. Trials were presented in one of two random orders, balanced across countries. The left-right position of the two test scenes was counterbalanced across trials. Infants in both countries saw exactly the same dynamic action scenes. What varied was whether the auditory materials were presented in English (US) or Mandarin (China).

##### Dialogue phase (17 s)

To begin each trial, we presented a video of two young women engaged in an animated conversation using infant-directed speech in the infants' native language. The goal of this phase was to capture infants' attention at the beginning of each trial. Table 3 presents one representative dialogue.

**Table 3:** **One representative Dialogue in English, Mandarin, and Pinyin transcription**.

	**English**	**Mandarin**	**Pinyin**
Person 1	Hey, you know what?	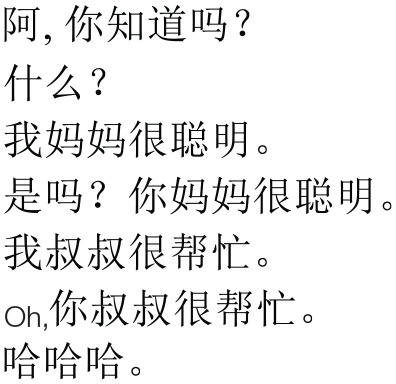	Ā, nǐ zhi¯ dào ma?
Person 2	What?		Shén me?
Person 1	My mother is very smart.		Wǒ mā mā hěn cōng míng.
Person 2	Oh really? Your mother is very smart.		Shì ma? Nǐ mā mā hěn cōng míng.
Person 1	My uncle is very helpful.		Wǒ shū shu hěn bang máng.
Person 2	Oh, your uncle is very helpful.		Oh, nǐ shū shu hěn bang máng.
Both	Hahaha.		Hahaha.

##### Familiarization phase (37.5 s)

For each trial, infants saw six different dynamic action scenes, each presented sequentially for 6.25 s. First, in the *exemplar scenes*, infants viewed four exemplars of a particular event, presented one at a time on alternating sides of the screen. In each, the same actor (e.g., a girl) performed the same action (e.g., petting) on one of four different objects of the same kind (e.g., four different toy dogs). The accompanying auditory materials (e.g. “Wow! Look what's happening here. Do you see that?”) were presented in infants' native language. Next, in the *contrast scenes*, infants viewed two dynamic scenes, presented one at a time in the center of the screen. Both involved the same actor as in the exemplar scenes (e.g., the girl). In the first contrast scene, this actor performed a new action on a novel object (e.g., the girl *drank* from a *cup*). On the accompanying audio, infants heard, e.g., “Uh-oh. Look at that.” Notice that this scene differed from the preceding familiarization scenes in two ways: it depicted a new action and a new participant object. This was a deliberate decision on our part: Our goal was (a) to reveal to infants that they would sometimes see scenes in which the object or the action could change, but (b) to insure that this scene could not, in itself, bias infants to focus on either the object or the action. In the second contrast scene, infants saw a familiar scene, selected randomly from the preceding familiarization scenes (e.g., the girl petting a toy dog). On the accompanying audio, infants heard, “Yay, look at that!” Here, our goal was to remind infants of the exemplar scenes they had viewed earlier. At the end of the final familiarization scene, the screen went blank and infants heard a bell chime as a star appeared at the center to orient infants' attention to the center. The star remained at the screen's center for 4 s, at which point the test phase began.

##### Test phase (13 s)

Finally, infants saw two new test scenes, presented simultaneously on either the right or left side of the screen. Both scenes included the familiar actor (e.g., the girl), but revealed a change in *either* the participant object *or* the action. In the New Object—Familiar Action scene, the actor used a new object (e.g., a pillow) to perform the now-familiar action (e.g., petting); in the New Action—Familiar Object scene, she performed a new action (e.g., kissing) with the now-familiar object (e.g., a dog). These test scene pairs appeared twice, once in a baseline period and again in a response period. See Arunachalam and Waxman (2011), Booth and Waxman (2009), Waxman et al. (2009) for other implementations of this design feature^3^.

In the *baseline* period (4 s), infants viewed the two test scenes, hearing, “Now look. They're different.” Because our goal in this period was to examine infants' baseline attention to the two test scenes, this linguistic phrase was intentionally designed to be neutral; it invites infants to observe the two test scenes without explicitly directing their attention to either one. Next, the screen went blank and infants heard a bell chime as a star appeared at the center. Our goal was to draw infants' visual attention to the center of the screen. The star remained at the screen's center (4 s) during which we introduced infants to the linguistic information they would also hear next in the response period (“Did you see it?”). After 4 s, the star disappeared, and the two test scenes re-appeared, each on the same side as it had appeared during baseline. In the *response* period (5 s), infants viewed these two test scenes, hearing once again, “Did you see it?” Our goal in this period was to examine infants' attention to the two test scenes. At the end of each trial, the screen went blank (0.33 s) before the next trial began. Table 4 presents the complete sequence of auditory information presented throughout the Familiarization and Test phase.

**Table 4:** **Language stimuli presented during Familiarization and Test in English, Mandarin, and Pinyin transcription**.

	**English**	**Mandarin**	**Pinyin**
Person 1:	*Wow! Look what's happening here*.	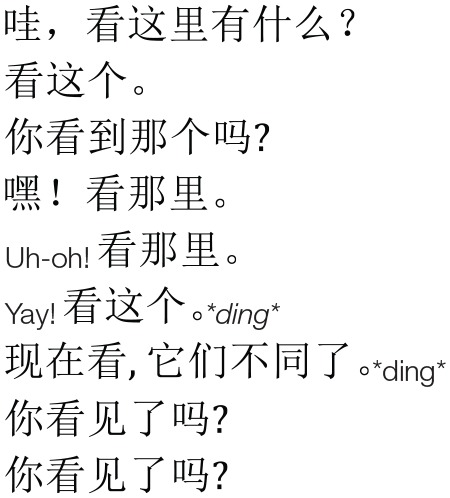	Wa, kàn zhè li yǒu shén me?
Person 2:	*Look at this*.		Kàn zhè ge.
Person 1:	*Do you see that?*		Nǐ kàn dào nàgè ma?
Person 2:	*Hey! Look there*.		Hey! Kàn nà lǐ.
Person 1:	*Uh-oh! Look at that!*		Uh-oh! Kàn nà lǐ.
Person 2:	Yay! Look at this! *ding*		Yay! Kàn zhè ge. *ding*
Person 1:	Now look. They're different. *ding*		Xiàn zài kàn, tā men bù tong le. *ding*
Person 2:	*Did you see it?*		Nǐ kàn jiàn le ma?
Person 2:	*Did you see it?*		Nǐ kàn jiàn le ma?

#### Data preparation

We defined two rectangular areas of interest (AOIs) of the same size, one to encompass each of the two test scenes (694 × 273 pixels). The eye-tracker automatically scored for validity each gaze location for each 60 hz sample for each pupil from each infant. When validity was high for both pupils (<=1 on Tobii's 0–4 scale), gaze location was computed as their average; when validity was high for only one pupil, gaze location was computed on that eye alone; when validity was low for both pupils, the sample was excluded from subsequent analyses. Next, for the purposes of statistical analysis, we focused on all gaze samples (both fixations and saccades) included within our two AOIs. We excluded any gaze samples that were outside of these two AOIs and any trials with excessive trackloss (defined as >2 *SD* of the total samples in the trial). Even with these exclusions, infants contributed data to more than 5 out of the 6 possible trials (US: *M* = 5.3, *SD* = 1.17; China: *M* = 5.8, *SD* = 0.52) and looked for the majority of each trial (US: *M* = 68% looking per trial, *SD* = 18%; China: *M* = 75% looking per trial, *SD* = 11%).

Before articulating our predictions, we underscore three design features that are essential to our logic. First, notice that in every scene, the participant object and the action in which it was engaged were inseparable in space: by definition, then, looking at a participant object (e.g., toy dog) necessarily entailed looking at the action (e.g., petting) and vice versa (see Rensink et al., 1997, for a description of advantages of this design feature and its implications for adults' attention to scenes). Second, recall that in the two test scenes, the participant object and action that were coupled during familiarization (e.g., dog, petting) were now deliberately uncoupled: one test scene involved a new object but maintained the familiar action and the other involved a new action but maintained the familiar object. Third, our predictions for the test phase follow the well-established logic underlying infants' visual preferences in tasks involving a period of familiarization followed by a test. In such tasks, after being familiarized sufficiently to one set of stimuli, infants typically exhibit a clear preference for looking at a novel stimulus (Roder et al., 2000; Colombo, 2002; Aslin, 2007)^4^.

#### Predictions

We predicted that infants from the US and China would be equally attentive during the familiarization phase, with no difference in the amount of time they spent looking at the scenes. Because during familiarization, the participant objects (e.g., dogs) and the actions in which they were engaged (e.g., petting) were fused together, infants' looking times to these scenes cannot, on their own, tease apart whether infants focused more on one or the other. Because participant objects and actions were uncoupled at test, performance in this phase can shed light on this matter. At issue, then, was how infants deployed their attention to the test scenes. Did their attention at test reflect the attentional proclivities of adults in their respective cultural communities? If by 24 months, infants' construals of dynamic scenes are influenced by the predominant attentional patterns of their respective cultures, then infants from the US and China should deploy their attention differently during test. If during familiarization, infants from the US focused more on the participant objects (e.g., dog) than the actions in which the objects were engaged (e.g., petting), then at test, they should prefer the scenes featuring the new object (New Object—Familiar Action scene, e.g., petting a *pillow*). By the same reasoning, if during familiarization, infants from China focused more on the actions (e.g., petting) than the particular participant objects (e.g. dog), then they should show the opposite trend, favoring the test scenes featuring the new action (New Action—Familiar Object scene, e.g., *kissing* a dog).

## Results

Familiarization trials. As predicted, there were no significant differences in infants' total accumulated looking time during familiarization, *p* = 0.084. This provides assurances that infants from the US and China were equally engaged in the task and attentive to the dynamic scenes.

**Figure 1:**
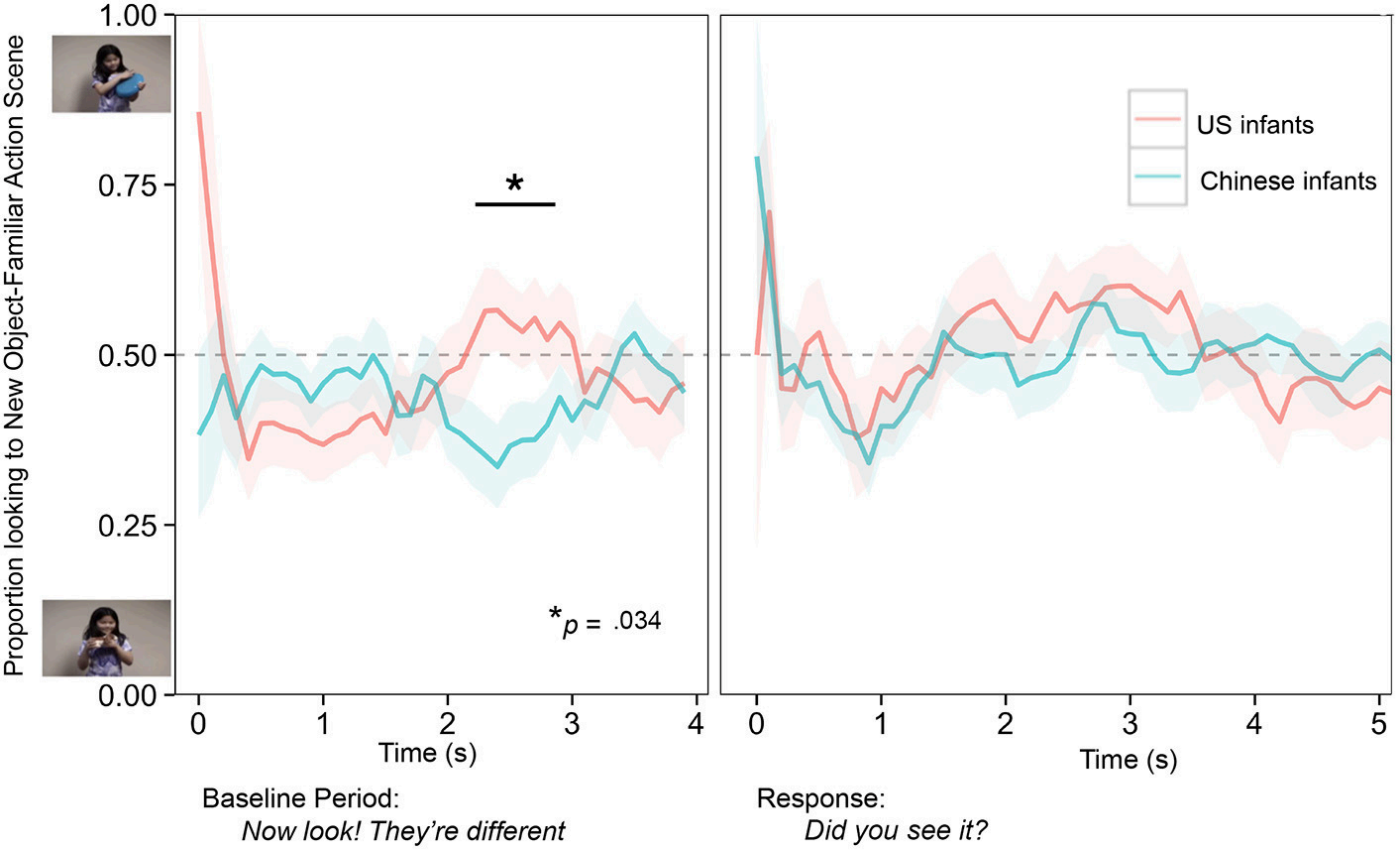
**The continuous timecourse (in seconds) of visual attention as it unfolds in real time over the entire test trial**. Shown here is the proportion of time infants devoted to the New Object—Familiar Action test scene (calculated as time looking at the New Object—Familiar Action scene divided by time looking at both the New Object—Familiar Action and the New Action—Familiar Object test scenes, with data aggregated across all trials for infants from the US and China. Proportions at 0.5 indicate equal attention to both scenes. Proportions near 1.0 indicate looking predominantly to the New Object -Familiar Action scene. Proportions near 0 indicate looking predominantly toward the New Action—Familiar Object scene. The shaded regions around each time-course line represents ±1 standard error of the mean (SEM); the asterisk denotes the significant effect during the baseline period revealed in the growth curve analysis (GCA). The horizontal line marks the segment in the baseline period when infants' attention in the two countries diverged (2.20–3.05 s, cluster-based permutation analysis). This segment occupies 21.2% of the baseline period and 8.5% of the entire test phase (baseline and response combined).

Test trials. To compare infants' patterns of attention during the test phase, when two different test scenes were available for inspection, we calculated for each infant and each trial, the mean proportion of looking time devoted to the New Object—Familiar Action (time looking toward the New Object—Familiar Action scene divided by the time looking toward both the New Object—Familiar Action and the New Action—Familiar Object test scenes). Note that for all analyses, we transformed all proportions using a logit transformation to avoid problems with analyzing raw proportions with linear models; (see Jaeger, 2008), for discussion. We report raw proportions in-text for interpretability. Figure 1 displays the continuous time-course of infants' looking behavior throughout the test phase.

A glance at this timeline reveals two observations. First, there appears to be striking similarity in the attentional patterns of infants raised in China and the US. Second, there appears to be a single exception to this overall convergence: During the baseline period, looking patterns in the two cultures appear to diverge briefly, with Chinese infants favoring the New Action—Familiar Object test scene and US infants showing the opposite pattern. These patterns are consistent with the possibility that by 24 months, infants' attention at test might reflect the attentional proclivities of adults in their respective cultures. To subject these observations to statistical test, we performed a complementary set of analyses using the *eyetrackingR* package (Dink and Ferguson, 2015).

First, we calculated the overall mean proportion of looking time that each infant devoted to the New Object—Familiar Action test scene, summing across each test period (baseline, response). We submitted this dependent measure to an analysis of variance with culture (2: China, US) as a between-participants factor and period (2: baseline, response) as a within-participants factor. There were no main effects, either for culture [*F*_(1, 38)_ = 0.025, *p* = 0.88] or period [*F*_(1, 38)_ = 2.20, *p* = 0.15], and no interaction between these factors [*F*_(1, 38)_ = 0.024, *p* = 0.63]. *Post-hoc t*-tests corroborated that infants from China and the US devoted the same overall proportion of attention to the New Object—Familiar Action scene (Baseline: *M*_CHINA_ = 0.44, *SD*_CHINA_ = 0.11; *M*_US_ = 0.45, *SD*_US_ = 0.15, *p* = 0.71; Response: *M*_CHINA_ = 0.48, *SD*_CHINA_ = 0.09; *M*_US_ = 0.49, *SD*_US_ = 0.11, *p* = 0.65).

Second, we analyzed the continuous time-course (rather than the overall mean proportion across a given period, as above) to identify any differences in how infants from China and the US directed their attention over real time as the dynamic scenes unfolded. To begin, we conducted a GCA using mixed-effects models (Baayen et al., 2008; Mirman et al., 2008) to ascertain whether there were any significant differences in the way US and Chinese infants' attention changed over time. This analysis required no prior assumptions about whether any such differences might emerge, when they might emerge in the test phase or the direction they might take.

To implement this GCA, we calculated—within each 100 ms bin across the entire test phase—the proportion of looking that each infant devoted to the New Object—Familiar Action scene. With this as our dependent measure, we modeled infants' looking over time in the baseline period and in the response period, using culture (China, US), time (using orthogonal polynomial time codes corresponding to linear, quadratic, cubic, quartic, and quantic growth trajectories, respectively), and the interaction between each time code and culture as fixed effects. We also included random intercepts and time slopes for both items and subjects (see Barr et al., 2013). The analysis revealed no significant effects during the response period, but revealed that during the baseline period, infants in the US and China did allocate their attention differently. We found a significant interaction between culture (China, US) and time (specifically, the cubic time parameter), β = 1.52, *SE* = 0.70, χ_(1)_ = 4.48, *p* = 0.034; see Figure 1. This interaction indicates that the attentional patterns of infants from the two cultures diverged significantly within a segment of the baseline period. There were no other main effects or interactions.

Third, we sought to pinpoint more precisely the timing of the significant cross-cultural difference identified in the GCA. To do so, we used a cluster-based permutation analysis (Maris and Oostenveld, 2007; see Oakes et al., 2013, for an example of this type of analysis in infant eye-tracking), using the proportion of looking that each infant devoted to the New Object—Familiar Action scene within each 25 ms bin across the baseline and response periods, respectively, as a dependent measure. The permutation analysis identified potential clusters by grouping together any adjacent bins in which there was any hint of an apparent separation between the two infant groups. At this point in the analysis, we imposed a conservative strategy to permit any adjacent bins that surpassed a *t* threshold corresponding to an α of.2 for this sample size; this relatively low threshold increased the number of adjacent clusters identified in the permutation analysis without increasing Type 1 error rates (Maris and Oostenveld, 2007). Finally, the cluster-based permutation analysis tested the likelihood that any of the identified adjacent clusters could have occurred by chance alone. This yielded a Monte Carlo *p*-value for each candidate cluster. In this way, this analysis permitted us to pinpoint the timing of the cross-cultural divergence.

This analysis revealed that infants' attention in the two cultures diverged from 2.20 to 3.05 s during the baseline period, *p* = 0.058, two-tailed. Importantly, this was the *only* divergence identified; all other candidate divergences were likely spurious, with *p*-values greater than 0.75; see Figure 1. This timing information tells us that attention in the two groups diverged as infants listened to the end of the phrase, “They're different.” Recall that this phrase was intentionally neutral; it did not explicitly direct infants' attention to either test scene. This divergence was modest in magnitude and short-lived; attention in both infant groups re-converged shortly thereafter (after roughly 1 s). Divergences of this duration are not uncommon in analyses of infant eye-tracking (Bunger et al., 2012; Arunachalam, 2013; Oakes et al., 2013; Ferguson and Waxman, 2014; Nordmeyer and Frank, 2014; Yin and Csibra, 2015).

Taken together, this suite of analyses documented broad cross-cultural similarities in infants' attention to objects and events throughout the test phase, with one single exception: looking patterns in the two cultures diverged significantly for a brief segment of the baseline period. As we discuss below, the direction of this divergence is consistent with the possibility that by 24 months, infants' construal of dynamic scenes may indeed be influenced subtly by the culture in which they are being raised.

## Discussion

The work presented here takes advantage of precise time-course analyses to provide new insight into how infants from the US and China deploy their visual attention while watching dynamic scenes as they unfold. At 24 months of age, infants from the US and China—infants who are on the threshold of learning words for objects and actions—show considerable commonalities in their attention to dynamic scenes. At the same time, however, we also identified an intriguing cross-cultural difference during the baseline period, when the patterns of attention of infants in the two cultures diverged significantly, albeit briefly. Moreover, the direction of this divergence is consistent with the possibility that by 24 months, infants' attention to dynamic scenes might be influenced by patterns characteristic of their respective cultures. Infants from China preferred the test scenes featuring the new action (New Action—Familiar Object scene, e.g., *kissing* a dog), as would be expected if during familiarization, they had focused more on the actions (e.g., petting) than the particular participant objects (e.g., dogs). In contrast, infants from the US showed the opposite pattern, attending to test scenes featuring the new object (Now Object—familiar Action scene, e.g., petting a *pillow*), as would be expected if during familiarization, they had focused more on the participant objects (e.g., dogs) than the actions in which the objects were engaged (e.g., petting). Clearly, 24-month-old infants from the US and China have a great deal in common when attending to dynamic scenes. But they may have also begun to “pick up” the attentional strategies characteristic of adults in their respective communities. The results reported here suggest that by the time they reach their second birthdays, infants may be on their way to becoming “native lookers” (see Werker et al., 2012, for the insight that in the realm of speech perception, infants increasingly become “native listeners”).

The current results underscore the value of conducting cross-cultural research with infants. After all, if we are to discover when culture-specific patterns of attention begin to shape our outlook on the world, we must set our sights on infants in the first years of life. However, these results offer only a first glimpse into how 24-month-olds' attention to objects and actions changes as dynamic scenes unfold in real time. Therefore, it will be important in future work to replicate these effects in the US and China, and to extend them in several ways. First, it will be important not only to replicate the cross-cultural divergence observed here, but also to identify more precisely the mechanisms underlying its timing. Interestingly, in the current experiment, the divergence occurred (between 2.20 and 3.05 s during the baseline period) while infants from both countries were listening to the end of the decidedly neutral phrase “Now look! They're different.” Although it is possible that this linguistic information somehow motivated infants from the two countries to allocate their attention differently, it is also possible that the timing reflects other attentional, cognitive or cultural processes. For example, it might reflect the processing time required to examine the two test scenes, to identify *how* the scenes differed, or to compare the scenes to locate the same particular object or action that they had seen in familiarization. A goal of future work will be to identify which of these processes, singly or in combination, best explain why infants' attention diverged at this specific point in the baseline period.

It will also be important to extend this paradigm to include infants from other cultures in which cultural differences in adults' attention to objects and events in scenes have also been documented (e.g., Japan, Korea). Another goal will be to extend the work to include younger infants to identify when the attentional differences that we have observed here begin to emerge. It will also be important to consider older toddlers and adults to ascertain whether the differences we have documented in this task at 24 months remain stable or become increasingly divergent with time. Finally, we plan to examine how infants deploy their attention in simpler tasks (e.g., including fewer familiarization trials or excluding the contrast trials).

In closing, the new evidence reported here, important in itself, provides a much-needed foundation for addressing longstanding debates about whether there are cross-linguistic differences in infants' early acquisition of nouns and verbs. To understand whether, and under what circumstances, novel words (either nouns or verbs) influence infants' attention to an ambient scene, we must first identify how infants deploy their attention to actions and objects when they are simply observing dynamic scenes as they unfold, and not simultaneously engaged in learning new words. There is wide agreement that across languages, infants acquire nouns (names for objects and object categories) more readily than verbs (names for events, actions and relations among objects) (Gentner, 1982; Tardif et al., 1997, 2008a; Imai et al., 2008). Some have suggested that this “noun advantage” may be attenuated for infants being raised in China, Korea or Japan as compared to those raised in the US (Waxman et al., 2013, provides a review). But whether and why this might be the case remains a topic of considerable debate that hinges on the role of experience in word learning. But addressing this requires that we next untangle how infants' various dimensions of experience—either linguistic experience, cultural experience, or both—shape the path of early lexical development. (Fernald and Morikawa, 1993; Waxman et al., 2013). As we move toward resolving this issue, the current evidence—which provides precise evidence of how 24-month-old infants from the US and China allocate their attentional resources, moment-by-moment, as they are observing dynamic scenes—will be essential. By documenting infants' performance in a neutral “resting state,” the evidence reported here will serve as a benchmark for specifying whether and how infants' moment-to-moment attention to objects and events in dynamic scenes might change when the very same scenes are described by either novel nouns or verbs. This is the focus of our current investigations with infants from the US and China.

### Conflict of interest statement

The authors declare that the research was conducted in the absence of any commercial or financial relationships that could be construed as a potential conflict of interest.

## Footnotes

^1^Although Katerelos and colleagues (Katerelos et al., 2011) documented infants' sensitivity to both objects and actions when no novel words were involved, their design did not pit infants' attention to new objects directly against their attention to new actions. Thus, it remains unclear whether infants' tendency, when given a choice, to direct their attention to objects vs. actions might itself vary as a function of the culture in which the infants were raised.

^2^Opaque glasses are standard practice in most infant research for the caregivers who accompany their infants in the experimental task; infants were not distracted by the glasses.

^3^Notice that in the current experiment, our instructions to the infants are neutral; they do not direct their attention to either one or the other test scene during either the baseline and response periods. In contrast, in prior implementations when we have introduced infants to novel nouns or verbs, the instructions were identical to those presented here during the baseline period, but did indeed direct infants attention during the response period (e.g., Noun conditions: “Where is the blick?” Verb conditions: “Where is he blicking something?”) For the sake of maintaining transparency, clarity and consistency across different implementations of this design, here we retain the terminology, describing the two periods of the test phase as baseline and response.

^4^Novelty preferences are ubiquitous, evident in tests of perceptual categories (e.g., after viewing a series of patches in one color, such as blue, infants prefer to look at a new color patch, such as green, over another blue one), object categories (e.g., after viewing several members of one object category, such as dogs, infants prefer to look at a member of a new category, such as a cat, over another dog) and action categories (e.g., after viewing several instances of one event category, such as waving, infants prefer to look at an instance of another action, such as tapping, over waving) (Bunger et al., 2012; Arunachalam, 2013; Oakes et al., 2013; Ferguson and Waxman, 2014; Nordmeyer and Frank, 2014; Yin and Csibra, 2015).
